# An atypical early-onset X-linked retinoschisis mimicking uveitis masquerade syndrome

**DOI:** 10.5935/0004-2749.202200103

**Published:** 2022

**Authors:** Alexandre de Carvalho Mendes Paiva, Fernando Henrique Flores Teixeira, Erika Moreira Carvalho, Nathalia Silva Santos, Ana Luiza Biancardi, André Luiz Land Curi

**Affiliations:** 1 Laboratory of Infectious Ophthalmology, Fundação Oswaldo Cruz, Rio de Janeiro, RJ, Brazil

Dear Editor,

Uveitis masquerade syndromes (UMSs) include systemic and ocular pathologies manifesting with intraocular infiltrating cells, but these are not due to immune-mediated or infectious uveitis entities^(1)^. Generally, UMS can be divided into neoplastic and non-neoplastic UMS. Hematologic malignancies and retinoblastoma represent examples of malignant conditions^(1)^. In contrast, non-malignant disorders, such as retinitis pigmentosa and retinal detachment, can be considered UMS^(2)^. Hereby we report a case of atypical early-onset X-linked retinoschisis (XLRS) mimicking UMS.

**Figure 1:**
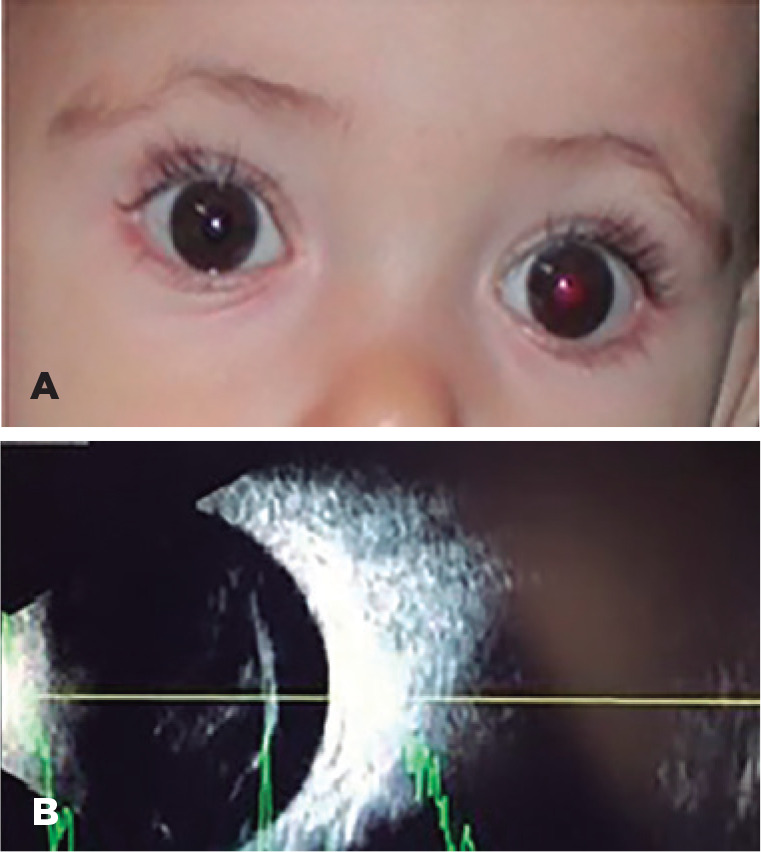
(A) Loss of red reflex in the right eye. (B) Right eye echography showing mobile vitreous opacities with medium to low reflectivity, thickened posterior hyaloid, attached retina, and the absence of chorioretinal thickening or calcification.

An 8-month-old male infant presented with a loss of red reflex and exotropia in the right eye (OD) for three months (Figure 1A). He was using sulfamethoxazole 400 mg/trimethoprim 80 mg as an oral pediatric suspension for four days because of a presumptive ocular toxoplasmosis diagnosis. His past medical, ocular, and mother’s prenatal care histories were unremarkable. The infant did not fix on or follow objects during OD evaluation. The exam identified poor fundus visualization owing to an intense yellow-colored vitreous opacity resembling vitritis without signs of a posterior lesion, such as “headlight in the fog”. Biomicroscopy and Perkins tonometry were unremarkable. The left eye (OS) exam was normal.

Ancillary tests were all negative, including *Toxoplasma gondii* serology. The patient underwent an echography that showed vitreous opacities, thickened posterior hyaloid, attached retina, and no signs suggesting retinoblastoma in the OD (Figure 1B). During the follow-up, the patient presented with a new episode of vitreous hemorrhage, and a UMS was considered.

**Figure 2:**
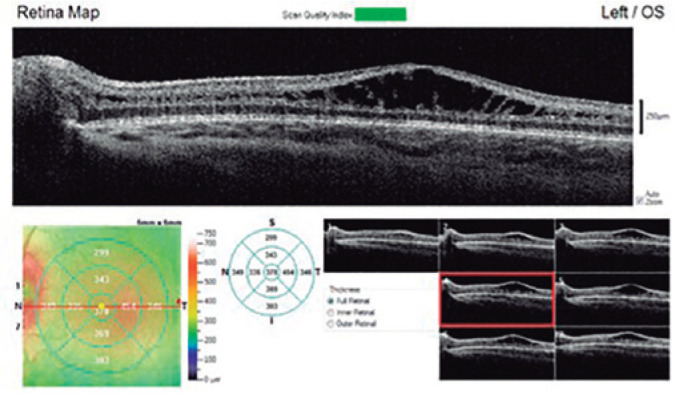
Macular optical coherence tomography of the left eye showing macular schisis with intraretinal separation between inner plexiform and inner nuclear retinal layers, extending beyond the foveal area.

Under general anesthesia, fluorescein angiography (FA) and optical coherence tomography (OCT) were performed. FA and OCT could not be performed in OD due to intense media opacity. FA was inconclusive in OS, revealing no dye leakage in the foveal area with window defects in the mid-periphery. However, OS OCT identified macular schisis with intraretinal separation between inner plexiform and inner nuclear retinal layers, extending beyond the foveal area (Figure 2).

Molecular genetic analysis of *RS1* was performed due to suspicion for the diagnosis of XLRS, detecting c.599 G>A (p.Arg200His) mutation; thus, a definitive diagnosis was made.

XLRS is a rare disorder, with a worldwide prevalence ranging from 1:5000 to 1:20000^(3)^. Its clinical hallmark is a spoke-wheel foveal schisis associated with a peripheral schisis in about 50% of cases^(3)^, predisposing to vitreous hemorrhage^(4)^. In the presented case, the hemorrhagic vitreous opacities were not absorbed, gaining a yellowish appearance. Therefore, it mimicked vitritis, leading to the misdiagnosis of uveitis. XLRS presents with low visual acuity at the age of 5-10 years^(4)^ and is usually symmetrical, but an asymmetry can be present, especially in cases with complications^(5)^. Few cases^3^ have been described in the first year of life. However, to the best of our knowledge, no one presented initially as a UMS.

The OS exam was normal, but OS OCT allowed diagnosing XLRS. Hence, the diagnosis was made based on OS assessment, although OD signs were the initial concern.

Molecular genetic studies identified mutations in *RS1* gene on chromosome Xp22, which codes for retinoschisin, a protein implicated in cellular adhesion and cell-to-cell interactions^(3)^. Despite the small size of the *XLRS1* gene, over 100 different disease-causing mutations are described. XLRS phenotypic variability does not appear to be dependent on the mutation type. The c.599 G>A (p.Arg200His) mutation was found in our patient, and a definitive diagnosis was made.

In conclusion, XLRS diagnosis may be challenging due to unusual manifestations in infant mimicking an UMS; hence, an accurate diagnosis can only be achieved following a complete multimodal assessment of both eyes, even in asymmetric cases when the initial examination of one eye appears normal.
